# Adherence to self-care recommendations and associated factors among adult heart failure patients in public hospitals, Addis Ababa, Ethiopia, 2021: cross-sectional study

**DOI:** 10.1186/s12872-022-02717-3

**Published:** 2022-06-17

**Authors:** Aemiro Baymot, Debela Gela, Tadesse Bedada

**Affiliations:** 1 Tikur Anbessa Hospital, Ababa, Ethiopia; 2 School of Nursing and Midwifery, College of Health Sciences, Addis Ababa University, Ababa, Ethiopia

**Keywords:** Adherence, Heart failure, Heart failure patient, Self-care recommendations, Addis Ababa, Ethiopia

## Abstract

**Background:**

Adherence to self-care recommendations in heart failure (HF) patients is essential to improve the patients’ quality of life, prevent hospital admission, and reduce mortality and morbidity. Nevertheless, poor adherence to self-care recommendations remains to be an extensive problem for HF patients. Thus, the aim was to assess adherence to self-care recommendations and associated factors among HF patients in public hospitals, Addis Ababa, Ethiopia, 2021.

**Methods:**

An institutional-based cross-sectional study was conducted among adult HF patients from February 15 to April 15, 2021, in five public hospitals, in Addis Ababa, Ethiopia. A total of 294 adult HF patients completed an interviewer-administered questionnaire in the Amharic language. The Revised HF Compliance Questionnaire was used to measure the adherence to self-care recommendations of HF patients. Data was collected using the Revised HF Compliance Questionnaire, the Japanese heart failure knowledge scale, the multidimensional scale of perceived social support, and the chronic diseases self-efficacy scale. Study participants were selected through a systematic random sampling technique. Data were entered into Epi-info version 7.1 and then exported to SPSS Version 25 for analysis. Descriptive and logistic regression analyses were performed and the statistical significance of associations between the variables was determined using ORs with 95% CI and *p*-values < 0.05.

**Results:**

Adherence to self-care recommendations among adult HF patients in public hospitals, in Addis Ababa, Ethiopia was 32.70%. Being female (AOR 4.66, 95% CI 1.58–13.67), patients who had high family monthly income (AOR 10.32, 95% CI 2.00–5.13), NYHA class III (AOR: 7.01, 95% CI 2.18–22.57) and class IV (AOR: 6.30, 95% CI 1.01–39.22), who had good self-efficacy (AOR 7.63, 95% CI 2.64–21.97), and who had good knowledge about HF (AOR 3.95, 95% CI 1.56–9.95) were more likely to have good adherence to self-care recommendations, *p-*value < 0.05.

**Conclusion:**

This study revealed that 32.70% of adult HF patients had good adherence to self-care recommendations. Factors associated with adherence to self-care recommendations of adult HF patients are sex, family monthly income, NYHA classification, self-efficacy, and knowledge about HF. Therefore, interventions focused on sex, family monthly income, NYHA classification, self-efficacy, and knowledge about HF are required to improve adherence to self-care recommendations of adult HF patients.

**Supplementary Information:**

The online version contains supplementary material available at 10.1186/s12872-022-02717-3.

## Background

Heart failure (HF) is one of the most common non-communicable diseases affecting a large number of people. The European Society of Cardiology (ESC) defined HF as “a clinical syndrome that occurs when the heart can’t keep up enough output.” It is characterized by symptoms like shortness of breath, persistent coughing or wheezing, ankle swelling, and fatigue that will be aimed at the subsequent signs like jugular venous pressure, pulmonary crackles, increased pulse rate, and peripheral edema [1, 2].

Globally, HF is a rapidly growing public health issue that affects greater than 37.7 million individuals and is the leading cause of hospitalization among adults and the elderly [3, 4]. In high-income nations like the USA, HF has affected over 6 million adult populations [5, 6]. In low- and middle-income countries (LMICs) HF is a serious burden to populations and health services, where it makes up a mean of 2.2% of hospital admissions and affects more men than women [7].

Although there is no population-based prevalence and incidence study in sub-Saharan Africa (SSA) including Ethiopia, the reported hospital prevalence studies indicate that HF is responsible for 9.4–42.5% of all medical admissions and in between 25.6 and 30.0% of admissions into the cardiac units. In SSA HF is a disease of young and middle age [8, 9]. Early-onset of HF in low economic regions especially in SSA is explained by the recurrent recurrence of rheumatic fever, rheumatic valvular heart diseases, congenital heart disease, and infective endocarditis [10–12]. The leading causes of heart failure in SSA are hypertensive heart disease, cardiomyopathy, rheumatic heart disease, and congenital heart diseases [13].

Adherence to HF self-care recommendations is non-pharmacological management of HF [14]. World Health Organization (WHO) defined self-care as “an ability of individuals, families, and communities to promote health, prevent disease, maintain health, and to cope or deal with illness and disability with or without the support of a healthcare provider” [15]. Self-care is a wide-ranging concept that also encompasses hygiene, nutrition, lifestyle, environmental factors, socioeconomic factors, and self-medication. HF patients' adherence to self-care recommendations requires adherence to medication, weight monitoring, sodium restriction, limitations of fluid, regular exercise, and appointment keeping [15–17].

Studies have demonstrated that good adherence to self-care recommendations in patients with HF is essential to improve the patients’ quality of life (QoL), prevent hospital admission, and reduce mortality and morbidity [16, 18]. Nevertheless, poor adherence to self-care recommendations remains to be an extensive problem for HF patients [19]. The proportion of good adherence to self-care recommendations among HF patients is 35.6% in the USA, 36.5% in Brazil, 48% in the Netherlands, 54.5% in Vietnam, 6.9% in Poland, 28% in Sudan, and 25.3% in Tanzania [19–25]. In Ethiopia, the proportion of good adherence to self-care recommendations among HF patients is 22.3% in Gondar and 28% in cardiac center Addis Ababa [5, 26]. A multitude of factors may affect good adherence to self-care recommendations among HF patients. These include demographic factors such as age, sex, marital status, religion, place of residence, educational status, occupation, and family monthly income [5, 20, 26–29], personal characteristics such as length of diagnosis, NYHA class of HF, comorbidity, history of hospitalization and number of admissions to a hospital [26, 27, 30–35], self-efficacy [36, 37], knowledge about HF [20, 26, 35, 38, 39] and social support [5, 21, 30, 31, 38, 40]. However, little is known about adherence to self-care recommendations among adult heart failure patients in Ethiopia. Thus, the aim was to assess adherence to self-care recommendations and associated factors among heart failure patients in public hospitals, Addis Ababa, Ethiopia, 2021.

## Method s and materials

### Study design, area, and population

**Fig. 1:**
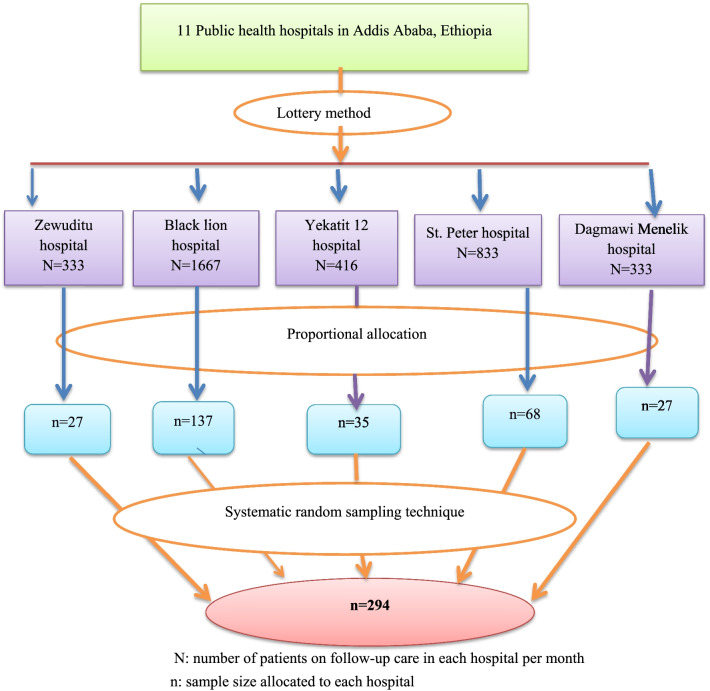
Schematic presentation of the sampling procedure for adult heart failure patients in public hospitals, Addis Ababa, Ethiopia, 2021(n = 294)

This cross-sectional study was conducted from February 15 to April 15, 2021, in five selected public hospitals from eleven public hospitals located in Addis Ababa, Ethiopia using the lottery method: Black Lion hospital, St, Peter hospital, Dagmawi Menelik hospital, Yekatit 12 hospital, and Zewuditu hospital. All adult (18 years or older) heart failure patients on follow-up care for at least 3 months were included except those who were critically ill at the time of data collection. The sample size was calculated using a single population proportion formula based on the assumptions of the 95% confidence level, 5% margin of error, and 22.3% population proportion taken from the study done in northwest Ethiopia [5]. A total of 294, with a 100% response rate was consented and recruited from five public hospitals in Addis Ababa (Additional file 1). The sample size (n) was proportionally allocated to each hospital depending on the number of patients on follow-up care in each hospital per month (N): Black Lion hospital (N = 1667/month, n = 137), St, Peter hospital (N = 833/month, n = 68), Dagmawi Menelik hospital (N = 333/month, n = 27), Yekatit 12 hospital (N = 416/month, n = 35), Zewuditu hospital (N = 333/month, n = 27). Each patient was finally selected using a systematic random sampling method with the patients’ follow-up registry serving as a sampling frame in each participating public hospital. To select each patient in each selected public hospital K internal was calculated depending on the number of patients on follow-up care per month (N) and proportionally allocated sample size (n) in each public hospital. For each public hospital K = N/n = 12 and K was between 1 and 12, then every 12 patients in the registry were selected from each public hospital with the first one determined using a lottery method (Fig. 1).

### Study variables

#### Dependent variable

The dependent variable was adherence to self-care recommendations.

#### Independent variables

The independent variables included socio-demographic factors (age, gender, marital status, religion, place of residence, educational status, occupation, family monthly income), personal factors (comorbidities, knowledge about heart failure, New York Heart Association (NYHA) functional class of the heart failure, hospitalization history), self-efficacy and social support.

### Data collection instruments and procedure

The Amharic version of the questionnaire was used for data collection. Data were collected by using a pre-tested and interviewer-administered questionnaire. First, the questionnaire was prepared in English language and then translated to Amharic (local language) and re-translated back to English to check for any inconsistencies. A pre-test was done on 5% of the study population (15 adult heart failure patients) at Tirunesh Beijing hospital in Addis Ababa, Ethiopia. The result of the pre-test was used to correct some unclear ideas and statements and the data was not incorporated into the main result. The questionnaire consisted of information on socio-demographic and personal characteristics, adherence to self-care recommendations, knowledge, self-efficacy, and social support. Data collection was in line with the patient’s follow-up dates. The data were collected by five trained professional nurses under the supervision of two supervisors and a principal investigator. Any misunderstanding during the interview process was resolved by discussion with the principal investigator. Further clinical data such as comorbidities, NYHA class, and hospitalization history were recorded from the patient’s medical record.

*The New York Heart Association (NYHA) classification of HF* grades patients' functional class from no restriction of exercise because of symptoms or Class I to Class IV where symptoms typical of HF, including fatigue and shortness of breath, are present even at rest [41].

The *Revised HF Compliance Questionnaire* was used to measure the adherence to self-care recommendations of heart failure patients [5, 22–24]. This tool contains six questions with a five-point scale (always = 4, mostly = 3, half of the time = 2, seldom = 1, never = 0). Patients were asked about (medication, low sodium diet, fluid restriction, and exercise) adherence level in the past week and about daily/three times per week/weight monitoring in the past month, and also about appointment keeping for the last 3 months. Cutoff points tool was classified as “*good adherence”* when they followed a recommendation ‘always’ or ‘mostly’ and *“poor adherence”* when they followed a recommendation half of the time, seldom, or never based on past studies [5, 22, 24]. The internal consistency of the measure was 0.88 (α) in this study.

The *Japanese heart failure knowledge scale* [42] was used to measure the patient’s knowledge about heart failure. This tool consists of three parts: first part general knowledge of patient about heart failure (1 question), the second part knowledge of patient about heart failure signs and symptoms (5 questions), third part knowledge of patient about heart failure-related self-care and treatment (8 questions). A total of fourteen questions were used to assess a patient’s knowledge about HF with a choice of ‘yes’, ‘no’, and ‘I don’t know.’ For each correct answer, 1 point is given and 0 was given for incorrect answers and I don’t know responses [5, 39]. The level of knowledge of patients about HF was classified as *“good”* for those who answered correctly greater than 75% of knowledge questions and as *“poor”* for those lower than 75% [5, 39]. The internal consistency of the scale was 0.75 [42]. The internal consistency of the measure was 0.79 (α) in this study.

The 12-item *Multidimensional Scale of Perceived Social Support (MSPSS)* [43] was used to measure the social support of patients. Items were scored on a 7-point Likert scale ranging from very strongly disagree (1) to very strongly agree (1). The score range is 12–84, with the scores above the mean indicating having good social support. The tool had internal consistency (α) of 0.87 [43] and 0.98 in this study.

The 6-item *chronic diseases self-efficacy scale* [44] was used to measure the self-efficacy of the patients. Originally each item contained a 10-point scale ranging from totally unconfident (1) to confident (10). The modified one has 5-point scale (1 = completely unconfident, 2 = unconfident, 3 = not sure, 4 = confident, 5 = totally confident). The scores ranged from 6 to 30, with the scores above the mean indicating having good self-efficacy. The reported internal consistency of the tool was 0.91 [44] and 0.96 in this study.

### Data analysis and processing

The collected data were coded, cleaned, and entered into Epi-info version 7.1 and then exported to SPSS Version 25 for analysis. Descriptive statistics were used to calculate frequencies, mean, and standard deviation of independent and dependent variables. A binary logistic regression analysis model was used to identify factors associated with adherence to self-care recommendations. Those variables with a *p*-value ≤ 0.200 in the bivariate logistic regression were entered into a multivariate logistic regression analysis. A multivariate logistic regression model was used to identify the association of independent variables and adherence to self-care recommendations. In multivariable logistic regression analysis, the statistical significance of associations between independent variables and adherence to self-care recommendations was determined using odds ratios with a 95% confidence interval and *p*-values < 0.05.

### Results

#### Socio-demographic characteristics of the participants

A total of 294 participants have participated with a response rate of 100%. Among the patients, 171(58.2%) were female, and the mean age was 45.03 with SD ± 18.10 years. More than half of the patients were married (n = 162, 55.11%) and lived in urban areas (n = 172, 58.50%). More than one-fourth of patients had the educational status of primary school (n = 81, 27.55%) and a family monthly income of ≤ 600 ETB (n = 82, 27.89%). The majority of patients were a follower of the orthodox Christian religion (n = 182, 61.90%) and 127 (43.20%) of patients were self-employed (Table 1).

**Table 1:** Socio-demographic characteristics of patients attending heart failure follow-up units of public health hospitals, Addis Ababa, Ethiopia, 2021 (n = 294)

Variables	Category	Frequency (n)	Percent (%)
Age in years	≤ 45	159	54.08
Mean (SD) = 45.03 ± 18.10	> 45	135	45.92
Sex	Male	123	41.84
	Female	171	58.16
Marital status	Single	90	30.61
	Married	162	55.11
	Divorced	24	8.16
	Windowed	18	6.12
Religion	Orthodox	182	61.90
	Muslim	61	20.75
	Protestant	42	14.29
	Catholic	9	3.06
Place of residence	Rural	122	41.50
	Urban	172	58.50
Educational status	Can’t read and write	46	15.65
	Can read and write	30	10.20
	Primary (1–8)	81	27.55
	Secondary (9–10)	47	15.99
	Preparatory (11–12)	41	13.95
	College and above	49	16.66
Occupation	Housewife	64	21.77
	Self-employed	127	43.20
	Student	25	8.50
	Governmental Employee	44	14.97
	Retired	18	6.12
	Others	16	5.44
Family monthly income in ETB	≤ 600	82	27.89
	601–1650	55	18.71
	1651–3200	39	13.27
	3201–5250	61	20.75
	5251–7300	27	9.18
	≥ 7301	30	10.20

ETB, Ethiopian birr

### Personal characteristics of the participants

More than half of the patients had comorbidities (n = 168, 57.14%) such as hypertension, chronic kidney disease, diabetes mallets, HIV, and ischemic heart diseases and 135(45.92%) of patients had a length of diagnosis of the disease between 5 to 10 years. Of all patients, 102 (34.69%) had NYHA class II heart failure, 146 (49.66%) had a history of hospitalization, and 89 (30.27%) were admitted to the hospital only one time (Table 2).

**Table 2:** Personal characteristics of patients attending heart failure follow-up unit of public hospitals in Addis Ababa, Ethiopia,2021 (n = 294)

Variables	Category	Frequency (n)	Percent (%)
Length of diagnosis	≤ 5 years	82	27.89
	5- 10 years	135	45.92
	> 10 years	77	26.19
NYHA class of heart failure	NYHA Class I	86	29.25
	NYHA Class II	102	34.69
	NYHA Class III	84	28.57
	NYHA Class IV	22	7.48
Comorbidity	No	126	42.86
	Yes*	168	57.14
History of hospitalization	No	148	50.34
	Yes	146	49.66
Number of admissions to hospital	No	148	50.34
	One time	89	30.27
	Two times	26	8.84
	> Two times	31	10.55

*Hypertension, Chronic kidney disease, Diabetes mallets, HIV, Ischemic heart diseases

### Knowledge, self-efficacy, and social support of the participants

Almost half of the patients, 144 (48.98%) had good knowledge about heart failure, half of the patients, 147(50.00%) had good self-efficacy and about 137 (46.60%) patients had good social support (Table 3).

**Table 3:** Knowledge, self-efficacy, and social support of patients attending heart failure follow-up units of public hospitals in Addis Ababa, Ethiopia, 2021. (n = 294)

Variable	Category	Frequency (n)	Percent (%)	Mean ± SD
Knowledge	Poor knowledge	150	51.02	9.94 ± 2.94
	Good Knowledge	144	48.98	
Self-efficacy	Poor self- efficacy	147	50.00	18.66 ± 7.86
	Good self-efficacy	147	50.00	
Social support	Poor social support	157	53.40	56.02 ± 19.68
	Good social support	137	46.60	

SD, Standard deviation

### Adherence to self-care recommendations of the participants

**Fig. 2:**
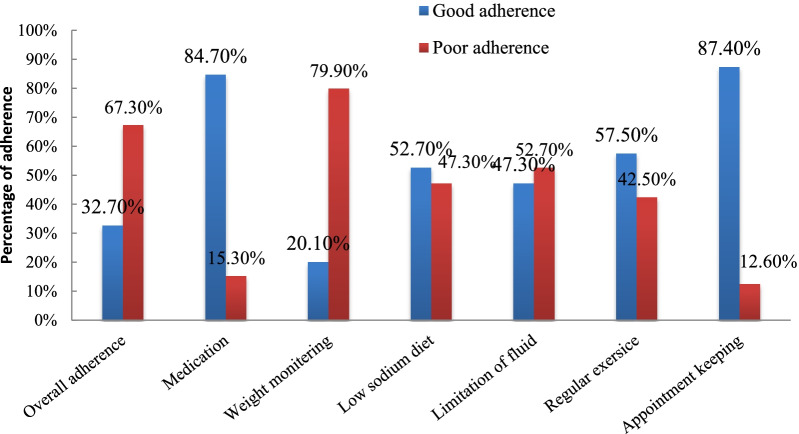
Level of adherence to self-care recommendations and its components among heart failure patients at public health hospitals in Addis Ababa, Ethiopia 2021 (n = 294)

Adherence to self-care recommendations among adult heart failure patients in public hospitals, in Addis Ababa, Ethiopia was 32.70%. Out of the six individual self-care recommendations, higher levels of adherence were noted in keeping appointments (n = 257, 87.40%) and taking prescribed medications as ordered by health care professionals (n = 249, 84.70%) (Fig. 2).

### Factors associated with adherence to self-care recommendations

In bivariate analysis, the covariates: sex, family monthly income; educational status, place of residence, history of hospitalization, number of admissions, length of diagnosis, comorbidity, NYHA class, self-efficacy, and knowledge about heart failure was associated with adherence to self-care recommendations among adult heart failure patients. In multiple logistic regression analysis, covariates: sex, family monthly income, NYHA class III and class IV, self-efficacy, and knowledge about heart failure were significantly associated with adherence to self-care recommendations at a 95% confidence interval and the model as a whole explained between 50.3% (Cox and Snell R square) and 70.1% (Nagelkerke R squared) of the variance in adherence to self-care recommendations, and correctly classified 88.1% of cases.

Being female was 4.66 times more likely to have good adherence to self-care recommendations than males (AOR: 4.66, 95% CI 1.58–13.67, *p* = 0.005). Patients who had high family monthly income were 10.32 times more likely to have good adherence to self-care recommendations compared to those who had lower family monthly income (AOR: 10.32, 95% CI 2.00–53.13), *p* = 0.005). Regarding NYHA classification, those patients with NYHA class III (AOR: 7.01, 95% CI 2.18–22.57, *p* = 0.001) and class IV (AOR: 6.30, 95% CI 1.01–39.22, *p* = 0.048) were 7.01 and 6.30 times more likely had good adherence to self-care recommendations, respectively compared to NYHA class I. Those patients who had good self-efficacy were 8 times more likely to have good adherence to self-care recommendations compared to those who had poor self-efficacy (AOR: 7.63, 95% CI 2.64–21.97, *p* = 0.000). Those patients who had good knowledge about heart failure were 3.94 times more likely to have good adherence to self-care recommendations compared to those who had poor knowledge about heart failure (AOR:3.95, 95% CI 1.56–9.95, *p* = 0.004) (Table 4).

**Table 4:** Factors associated with adherence to self-care recommendations of patients attending heart failure follow-up units of public hospitals in Addis Ababa, Ethiopia, 2021 (n = 294)

Variables	Category	Adherence to Self-care		COR (95% CI )	AOR (95% CI )	*p*-value	Collinearity Statistics	
		Good	Poor				Tolerance	VIF
Sex	Male	27	96	1	1		0.91	1.09
	Female	69	102	2.41(1.42–4.06)	4.65(1.58–13.67)	0.005		
Marital status	Single	18	72	1	1		0.93	1.08
	Married	71	91	3.12(1.70–5.70)	1.01(0.31–3.43)	0.939		
	Divorced	4	20	0.8(0.24–2.63)	0.93(0.13–6.23)	0.941		
	Windowed	3	15	0.8(0.20–3.06)	0.56(0.05–5.22)	0.608		
Place of residence	Rural	24	98	1	1		0.89	1.13
	Urban	72	100	2.94(1.71–5.04)	1.49(0.58–3.81)	0.402		
Educational status	Can’t write & read	8	38	1	1		0.72	1.39
	Can write & read	5	25	0.95(0.27–3.23)	2.43(0.30–9.53)	0.405		
	Primary (1–8)	23	58	1.88(0.76–4.64)	1.23(0.26–5.67)	0.795		
	Secondary (9–10)	15	32	2.23(0.83–5.92)	1.63(0.33–0.327)	0.556		
	Preparatory (10–12)	24	17	6.71(2.50–7.93)	1.62(0.28–9.342)	0.587		
	College and above	21	28	3.56(1.37–0.20)	1.67(0.25–0.783)	0.592		
Family monthly income in ETB	≤ 600	7	75	1	1		0.65	1.55
	601–1650	11	44	2.67(0.96–0.41)	1.75(0.41–7.15)	0.456		
	1651- 3200	11	28	4.2(1.48–11.93)	7.05(1.37–6.17)	0.019		
	3201- 5250	26	35	7.96(3.15–0.09)	3.52(0.93–1.26)	0.630		
	5251–7300	17	10	18.2(6.06–15.00)	10.32(2.00–53.13)	0.005		
	≥ 7301	6	24	60.7(14.22–25.18)	11.73(1.62–84.54)	0.015		
Comorbidity	Yes	70	98	2.75(1.61–4.66)	0.69(0.23–2.09)	0.519	0.89	1.13
	No	26	100	1	1			
NYHA classification	Class I	13	73	1	1		0.86	1.16
	Class II	24	78	0.73(0.81–3.64)	1.64(0.53–5.05)	0.386		
	Class III	53	31	9.6(4.59–20.08)	7.01(2.18–22.57)	0.001		
	Class IV	6	16	2.11(0.69–6.37)	6.3(1.01–39.22)	0.048		
Self-efficacy	Good self-efficacy	82	65	11.9(6.32–22.72)	7.63(2.64–21.97)	0.000	0.59	1.69
	Poor self-efficacy	14	133	1	1			
Social support	Good social support	73	64	6.64(3.81–11.57)	2.12(0.85–5.24)	0.105	0.62	1.60
	Poor social support	23	134	1	1			
Knowledge	Good knowledge	78	66	8.67(4.79–15.65)	3.95(1.56–9.95)	0.004	0.57	1.75
	Poor knowledge	18	132	1	1			

The chi-square value for the Hosmer–Lemeshow Test is 8.810 with a *p*-value of 0.359

*p*-value < 0.05: statistically significant; CI Confidence Interval; COR: Crude Odd Ratio; AOR: Adjusted Odd Ratio

## Discussion

This study explored the adherence to self-care recommendations and associated factors among adult heart failure patients in public hospitals, in Addis Ababa, Ethiopia, and found that 32.70% of adult heart failure patients in public hospitals, in Addis Ababa had good adherence to self-care recommendations. This result shows a low level of adherence to self-care recommendations among adult heart failure patients. The proportion of good adherence to self-care recommendations among adult heart failure patients in this study was higher than that of a study done in Gondar (22.3%) and in the cardiac center in Addis Ababa (28%), Ethiopia [5, 35] and other countries like Poland (6.9%), Tanzania (25.3%), Sudan (28%) [23–25]. However, its proportion was lower than that of a study done in other countries like the USA (35.6%), Brazil (36.5%), Netherlands (48%), and Vietnam (54.5%.) [19–22]. This might be related to the fact that there is a difference in the study population, study design, and measurement tools. For instance, a study done in the USA was among heart failure patients residing in urban areas, but this study was done among heart failure patients residing in both urban and rural areas. Most of the studies done in developed countries was using a prospective cohort study design, however, this study was done using a cross-sectional study design. Thus, prospective and experimental studies are warranted.

This study identified that sex, family monthly income, NYHA class, self-efficacy, and knowledge about heart failure have an association with adherence to self-care recommendations of adult heart failure patients. This study revealed that heart failure patients who were females had good adherence to self-care recommendations compared to males. This finding was supported by a study done in California [28]. This might be related to the fact that females are more involved in self-care in their work than males. Nevertheless, the finding of this study is in contrast to the study done in Gondar, Ethiopia that found a positive association between male sex and adherence to self-care recommendations [5]. This discrepancy might be due to cultural differences between the two study populations. Therefore, male adult heart failure patients need special emphasis when designing interventions aimed at improving adherence to self-care recommendations for heart failure patients.

This study shows that heart failure patients who had higher family monthly income had good adherence to self-care recommendations compared to those who had lower family monthly income. This implies that heart failure patients who had higher family monthly income may easily fill materials like medication, food, and other related materials, may have more access to social media like Television, Telegram and may have the family doctors that help them to improve adherence to self-care recommendations. However, this finding was different from some other studies that found no significant association between adherence to self-care recommendations and monthly income [5, 24]. This difference might be due to the difference in the study area and study design. Therefore, heart failure patients with lower family monthly incomes need special emphasis when designing and implementing interventions aimed at improving the adherence to self-care recommendations of this population group. This may involve the provision of economic support through established safety net programs in the community.

Our study shows that heart failure patients who had NYHA class III and class IV had good adherence to self-care recommendations compared to those who had class I. This result was similar to the other studies done in Ethiopia [32, 34]. This might be related to the fact that heart failure patients who had NYHA class I didn’t have any signs and symptoms of HF and didn’t worry about the disease. Nonetheless, in the case of NYHA classes III and IV, the symptoms are more severe and the patients are aware that they have a disease and adhere to the pharmacological and non-pharmacological treatments. Therefore, heart failure patients with NYHA class I need special attention when designing and implementing interventions aimed at improving the adherence to self-care recommendations.

This study also shows that heart failure patients who had good self-efficacy had good adherence to self-care recommendations compared to those who had poor self-efficacy. Some studies reported that to improve heart failure patients’ adherence to self-care recommendations, better to focus on patient mental development to increase self-efficacy [36, 37]. This might be related to the fact those heart failure patients who have good self-efficacy believe that taking medication properly will bring cure or health, and can organize and execute the courses of action required to produce given attainment.

In this study, another modifiable factor associated with adherence to self-care recommendations was knowledge about heart failure. Those participants who had good knowledge about heart failure had good adherence to adherence to self-care recommendations than those who had poor knowledge about heart failure. This finding is congruent with the other studies done in Vietnam, and Ethiopia [5, 20, 26, 35, 39]. This is might be related to the fact that those patients who know well about heart failure, its signs and symptoms, and self-care behavior may perform more self-care than those patients who didn’t know. Prospective studies are also needed to determine the effects of knowledge about heart failure on adherence to self-care recommendations among adult heart failure patients.

## Limitation of the study

This study has a couple of limitations. First, use of an interviewer-administered structured questionnaire for data collection. Using this method to identify adherence to self-care recommendations and associated factors among adult heart failure patients might involve some risk, though qualitative interviews can let participants liberally highlight their concerns and obstacles concerning adherence to self-care recommendations. Second, the use of cross-sectional design does not allow inferring causality. Prospective and experimental studies are warranted. Third, the study did not include heart failure patients who were attending follow-up in private hospitals.

## Conclusion

This study revealed that 32.70% of adult heart failure patients had good adherence to self-care recommendations. Factors associated with adherence to self-care recommendations of adult heart failure patients are sex, family monthly income, NYHA classification, self-efficacy, and knowledge about heart failure. Therefore, interventions focused on sex, family monthly income, NYHA classification, self-efficacy, and knowledge about heart failure are required to improve adherence to self-care recommendations of adult heart failure patients. Prospective studies are also needed to determine the effects of knowledge about heart failure on adherence to self-care recommendations among adult heart failure patients.

## Supplementary Information

**Additional file 1.** Sample size and sampling procedure.

## Data Availability

Datasets used and/or analyzed during the current study are available from the corresponding author on reasonable request.

## Abbreviations

AOR: Adjusted odds ratio
CI: Confidence interval
ESC: European Society of Cardiology
ETB: Ethiopian Birr
HF: Heart failure
HIV: Human immunodeficiency virus
LMICs: Low and middle-income country
MSPSS: Multidimensional scale of perceived social support
NYHA: New York Heart Association
SSA: Sub Saharan Africa
QoL: Quality of life
TASH: Tikur Anbessa Specialized Hospital
USA: United States America
WHO: World Health Organization
