# Supplementary material for: Adherence to self-care recommendations and associated factors among adult heart failure patients in public hospitals, Addis Ababa, Ethiopia, 2021: cross-sectional study
Source: BMC Cardiovasc Disord. 2022 Jun 17;22:275. doi: 10.1186/s12872-022-02717-3 (PMC9206252; doi:10.1186/s12872-022-02717-3)
Supplement: Supplementary file 1 — Additional file 1. Sample size and sampling procedure. [file 12872_2022_2717_MOESM1_ESM.docx]

**Adherence to self-care recommendations and associated factors among adult heart failure patients in public hospitals, Addis Ababa, Ethiopia, 2021. Cross-sectional study**

**Authors**

Aemiro Baymot (MSc)^1^, Debela Gela (Assistant Professor)^2^, Tadesse Bedada (Lecturer, PhD. fellow)^3^

**Corresponding author: Debela Gela**

**Short Title: Adherence to self-care recommendations among adult heart failure patients**

**Sample size calculation for adult heart failure patients in public hospitals, Addis Ababa, Ethiopia, 2021.**

The sample size was calculated using the single population proportion formula. Considering a population of the estimated proportion of self-care adherence in patients with heart failure assumed to be 22.3%, level of confidence of 95%, and margin of error 5%.

Sample size (n) =(Zα_/2_)^2^×p(1-p)

d^2^

n= (1.96)^2^ X0.223X0.777

(0.05)^2^

n ~267

Where p – population proportion of patients who adhere to self-care practices

d = 0.05 (5% margin of error)

Z = critical value for normal distribution at 95% confidence level which equals to 1.96 (z value at α =0.05)

n- Required sample size

Adding a non-response rate of 10%, the final required sample size is 294.
